# Rebamipide Induces Hair Regeneration Through EP4-Driven Lipid Metabolism Remodeling

**DOI:** 10.3390/ijms262010132

**Published:** 2025-10-18

**Authors:** Chenjie Feng, Hao Dong, Dongyue Jiang, Yuan Gao, Xinyue Gu, Weiwei Diao, Ying Zhou, Dayang Xu, Ruixin Li, Liang Wu

**Affiliations:** 1 New Drug Screening and Pharmacodynamics Evaluation Center, State Key Laboratory of Natural Medicines, China Pharmaceutical University, Nanjing 210009, China; 17351013360@126.com (C.F.); zgmfx666s@outlook.com (H.D.); markjiang1998@hotmail.com (D.J.); zsgydyx@163.com (Y.G.); xdy3108236416@163.com (D.X.); lrxx0201@163.com (R.L.); 2 Department of Biology, Johns Hopkins University, Baltimore, MD 21218, USA; xgu18@jhu.edu; 3 School of Traditional Chinese Pharmacy, China Pharmaceutical University, Nanjing 210009, China; diao458820@icloud.com (W.D.); zhouying2900@163.com (Y.Z.)

**Keywords:** hair regeneration, rebamipide, lipolysis, dedifferentiation, EP4 receptor

## Abstract

Alopecia is a highly prevalent hair loss disorder characterized by an abnormality in hair cycling. Induction of autophagy and secretion of growth factors by adipocyte precursors are sufficient to activate quiescent hair follicles, yet therapies targeting these processes remain limited. Here, we identify rebamipide—a drug originally intended for gastric ulcer treatment—as a promising candidate for hair regeneration by modulating dermal adipocyte metabolism. Topical rebamipide treatment induces autophagy and adipose triglyceride lipase (ATGL)-mediated lipolysis in dermal adipocytes. Using primary culture systems, we demonstrate that rebamipide-driven lipolysis triggers adipocyte dedifferentiation, activating hair follicle stem cells (HFSCs) via elevated platelet-derived growth factor (PDGF) levels. Mechanistically, computer simulations and target validation experiments confirm that rebamipide directly binds to the prostaglandin E receptor EP4, triggering PI3K/ERK-dependent autophagy and lipolysis. Collectively, our findings highlight EP4 as a novel therapeutic target for hair loss and position rebamipide as an agent that couples lipid metabolism remodeling with HFSC activation.

## 1. Introduction

Hair loss has become a global health concern with progressively increasing incidence. In China, the prevalence of androgenetic alopecia (AGA) reaches 21.3% in males and 6.0% in females, which is even higher in Caucasians [1,2,3]. Hair loss is characterized by the failure of hair follicles (HFs) to enter the telogen–anagen transition, leading to miniaturization and degeneration of the HFs [4]. The only two Food and Drug Administration (FDA)-approved drugs for hair loss are minoxidil and finasteride. Despite their clinical effectiveness, both require chronic usage and may cause significant side effects [5]. Given the health and social impacts of this condition and the limitations of current treatments, there is an urgent need to develop safer and more effective therapies for hair regeneration.

During anagen, HFs extend deep into the dermis, closely interacting with dermal white adipose tissue (dWAT). These interactions occur throughout various stages of the hair cycle. During anagen, HFs stimulate dWAT hypertrophy and hyperplasia, while in telogen, mature adipocytes secrete bone morphogenetic proteins (BMPs) that maintain hair follicle stem cells (HFSCs) quiescence and suppress telogen–anagen transition (TAT) entry [6,7]. Meanwhile, adipocyte precursors secrete high levels of platelet-derived growth factor subunit-α (PDGFα), which activates resting HFs and drives hair regeneration [8,9]. Since adipocyte precursors and mature adipocytes can undergo conversions [7,10], it is highly possible that dedifferentiation of dermal adipocytes contributes to TAT. Recent advances in adipocyte biology have revealed that mature adipocytes can dedifferentiate into proliferative progenitor-like cells, termed Dedifferentiated Fat (DFAT) cells, through ceiling culture methods [11]. These DFAT cells exhibit multipotency and can redifferentiate into adipocytes under appropriate conditions, serving as a valuable model for studying adipocyte plasticity.

Hair follicles harbor a heterogeneous population of stem cells residing in distinct niches, including the bulge, hair germ, isthmus, and infundibulum, each with unique molecular and functional features that regulate hair cycling and skin homeostasis [12]. Among these, bulge stem cells (BuSCs) represent a well-characterized, slow-cycling, and multipotent population that is essential for long-term hair follicle regeneration and cyclical growth. In this study, we isolated and cultured BuSCs because they are the quintessential HFSC population with a well-defined identity and regenerative capacity, making them an ideal model for studying adipocyte–HFSC crosstalk in hair cycle regulation.

Autophagy is an evolutionarily conserved lysosomal degradation pathway that plays a critical role in hair regeneration. Autophagy-activating molecules, such as α-ketoglutarate, metformin, rapamycin [13], isoquercitrin [14] and limonin [15], have been shown to initiate anagen in telogen HFs and stimulate hair growth. Notably, autophagy in adipocytes regulates lipid metabolism and energy homeostasis by degrading lipid droplets and triglycerides [16], as well as inducing adipocyte dedifferentiation [17].

Rebamipide, an FDA-approved gastroprotective agent, promotes ulcer healing [18] and mitigates alcohol-induced gastric mucosal damage via autophagy activation [19]. Pharmacokinetic studies show that rebamipide is rapidly absorbed (T_max_ = 2.4 h) with a short half-life (1.9 h) and minimal systemic exposure (<5% bioavailability), which accounts for its localized efficacy in gastrointestinal tissues [20]. Mechanistically, it enhances prostaglandin E2 production and scavenges reactive oxygen species [18]. Due to its low bioavailability, rebamipide has been repurposed for topical applications. Clinical trials have demonstrated its efficacy in treating dry eye [21] and oral mucositis [22]. Additionally, it ameliorates atherosclerosis and obesity phenotypes by regulating lipid metabolism and suppressing adipogenesis [23,24]. However, the effects and molecular mechanisms of rebamipide in hair cycle regulation remain unknown.

To systematically investigate the role of rebamipide in hair regeneration, this study integrated murine models, primary adipocyte cultures, molecular biology assays, and computational approaches. We first established its efficacy in stimulating hair regrowth through topical applications and then explored underlying mechanisms, focusing on metabolic reprogramming in dermal adipocytes. We further examined how these metabolic changes promote adipocyte dedifferentiation and paracrine PDGFα secretion. Molecular docking and experimental target validation identified binding to the EP4 receptor as the mechanistic basis of rebamipide’s activity. These findings establish rebamipide as a promising new candidate for hair loss treatment, acting through adipocyte metabolic remodeling and subsequent HFSC activation.

## 2. Results

### 2.1. Topical Treatment with Rebamipide Induces Hair Regeneration

**Figure 1:**
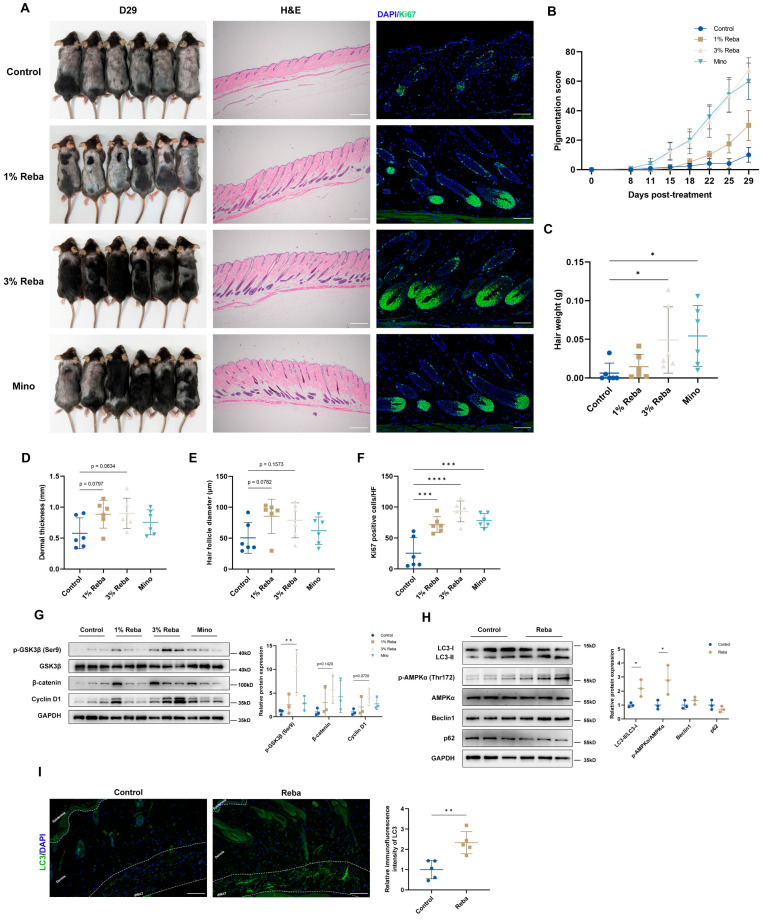
Rebamipide stimulates autophagy to promote hair regeneration in vivo. (**A**) Seven-week-old male C57BL/6 mice were shaved and treated topically with vehicle control, 1% rebamipide (Reba), 3% rebamipide, or 2% minoxidil (Mino) for 29 days. Mice were sacrificed for subsequent analyses. Photographs were taken on day 29 post-treatment. Melanin deposition in the skin served as an indicator of anagen onset. H&E staining of skin sections revealed that rebamipide treatment increased dermal thickness, hair bulb size, and hair shaft elongation compared to the control group. Ki67 immunofluorescent staining revealed enhanced cellular proliferation in the hair bulbs of rebamipide-treated mice. Nuclei were stained with DAPI (blue). Scale bars represent H&E staining, 500 μm; Ki67 staining, 100 μm. (**B**) Quantification of melanin pigmentation appearance (n = 6 for each group). (**C**) Quantification of regrown hair weight to assess hair regrowth (n = 6 for each group). (**D**) Quantification of dermal thickness in the dorsal skin (n = 6 for each group). (**E**) Quantification of hair follicle diameter (n = 6 for each group). (**F**) Quantification of Ki67-positive cells per hair follicle (n = 6 for each group). (**G**) Western blot analysis of key β-catenin pathway components in dorsal skin (n = 3 for each group). GAPDH served as a loading control. (**H**) Western blot analysis of autophagy-related proteins in telogen-phase skin of mice treated with 3% rebamipide for 7 days (n = 3 for each group). (**I**) Immunofluorescent staining of LC3 in the dermal white adipose tissue (dWAT) layer of the dorsal skin treated with 3% rebamipide for 7 days to assess autophagy activation (n = 5 for each group). Nuclei were stained with DAPI (blue). Scale bars, 100 μm. Data are presented as means ± standard deviation (SD), * *p* < 0.05, ** *p* < 0.01, *** *p* < 0.001, and **** *p* < 0.0001 compared with the control group.

We first evaluated the hair regeneration-promoting effects of rebamipide in a telogen-phase mouse model. The results showed that rebamipide had a dose-dependent effect on accelerating hair regrowth. The 3% rebamipide group exhibited increased pigmentation scores and regrown hair weight, with effects comparable to 2% minoxidil (Figure 1A–C). In contrast, the control group showed minimal pigmentation until day 29. With anagen development, HFs grow downward into the deep dermis and increase in size [25]. The rebamipide-treated groups exhibited increased dermis thickness and hair bulb size compared to the control group (Figure 1D,E). Immunofluorescent (IF) staining for the proliferative marker Ki67 revealed more proliferating cells around the hair matrix in treated groups (Figure 1F). Western blot (WB) analysis confirmed activation of the Wnt/β-catenin pathway, a key regulator of hair growth [26]. Rebamipide significantly upregulated the expression of phospho-glycogen synthase kinase 3β (p-GSK3β) (Ser9), β-catenin and cyclin D1 (Figure 1G), demonstrating that rebamipide activates the β-catenin pathway and induces hair regeneration.

As autophagy is essential for activating quiescent telogen HFs, we investigate the effect of rebamipide on autophagy induction. To this end, a separate cohort of mice was treated with 3% rebamipide for 7 days to assess its effects before the onset of anagen. Quantitative immunoblotting was employed to evaluate autophagic activity. The conversion of LC3-I to its lipidated form, LC3-II (reflected by an increased LC3-II/LC3-I ratio), is a well-established marker of autophagosome formation. Additionally, the degradation of the autophagy receptor p62/SQSTM1 indicates successful autophagic flux [27]. Our results revealed a 2.2-fold increase in the LC3-II/LC3-I ratio (*p* < 0.05) along with a 30% reduction in p62/SQSTM1 (Figure 1H). Furthermore, rebamipide enhanced AMP-activated protein kinase (AMPK) phosphorylation, indicating AMPK-mediated autophagy induction. IF staining showed increased LC3 puncta, which represent autophagic vesicles, and enhanced fluorescence intensity in the dWAT layer, suggesting rebamipide-induced autophagy activation in dWAT (Figure 1I).

### 2.2. Rebamipide Induces Dermal Adipose Tissue Lipolysis

**Figure 2:**
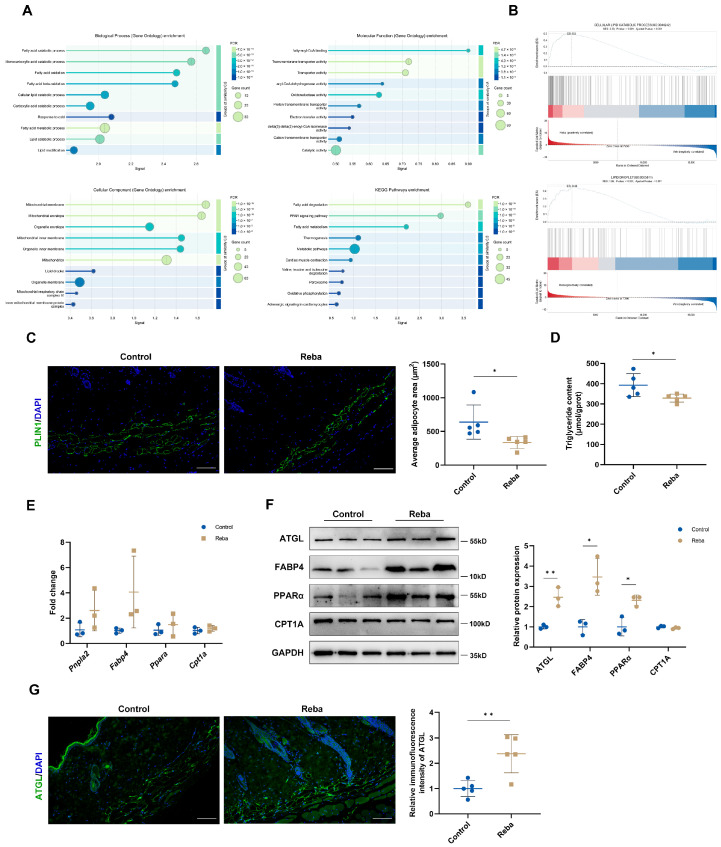
Rebamipide induces lipolysis in dermal adipose tissue in vivo. (**A**) Dorsal skin tissues from telogen-phase mice treated topically with rebamipide for 7 days were subjected to RNA-seq analysis and molecular biology assays (n = 3 for each group). Upregulated genes identified by hierarchical clustering were selected for functional enrichment analysis using Gene Ontology (GO) and Kyoto Encyclopedia of Genes and Genomes (KEGG) databases. (**B**) Gene Set Enrichment Analysis (GSEA) of cellular lipid catabolic process and lipid oxidation. (**C**) Representative PLIN1 immunostaining images of dWAT and quantification of adipocyte size (n = 5 for each group). Nuclei were stained with DAPI (blue). Scale bars, 100 μm. (**D**) Quantification of triglyceride (TG) content in dorsal skin tissue homogenates (n = 5 for each group). (**E**) Expression of lipolysis-related genes in telogen skin measured by quantitative PCR (qPCR) (n = 3 for each group). (**F**) Western blot analysis of lipolysis-related proteins in telogen skin (n = 3 for each group). (**G**) Representative ATGL immunostaining images showing enhanced lipolytic response in rebamipide-treated dWAT adipocytes (n = 5 for each group). Nuclei were stained with DAPI (blue). Scale bars, 100 μm. Data are presented as means ± SD, * *p* < 0.05 and ** *p* < 0.01 compared with the control group.

To elucidate the underlying mechanism of rebamipide in hair regeneration, we conducted RNA-seq on the dorsal skin of C57BL/6 mice after 7 days of treatment. Hierarchical clustering identified 2079 differentially expressed genes (DEGs) that were upregulated by rebamipide treatment. Since the analysis was performed on full-thickness skin, the large number of DEGs reflects responses from multiple cell types. We selected the top 200 DEGs for functional enrichment pathway analysis. Gene Ontology (GO) enrichment analysis revealed upregulated pathways involved in the fatty acid catabolic process, fatty-acyl-CoA binding, and mitochondrion (Figure 2A). Kyoto Encyclopedia of Genes and Genomes (KEGG) analysis showed that rebamipide treatment led to enrichment of genes associated with fatty acid degradation. This result was further supported by Gene Set Enrichment Analysis (GSEA), which similarly revealed significant enrichment in lipid catabolism and oxidation pathways (Figure 2B). This pattern strongly indicates that the changes in lipid and energy metabolism are primarily driven by the abundant dermal adipocytes. These findings highlight the broad impact of rebamipide on lipid metabolism remodeling in the skin, prompting further investigation into its effects on dWAT.

To evaluate the effects of rebamipide on lipid metabolism, we performed perilipin 1 (PLIN1) IF staining to measure the size of lipid droplets in adipocytes. Rebamipide significantly reduced adipocyte size (Figure 2C) and skin triglyceride (TG) content (Figure 2D), indicating enhanced lipolysis in the skin. Quantitative PCR (qPCR) and WB analysis (Figure 2E,F) further confirmed increased expression of *Pnpla2*/ATGL, which catalyzes the initial step in TG hydrolysis [28]. This result was supported by ATGL enrichment in dermal adipocytes, as shown by IF staining (Figure 2G). Additionally, increased expression of fatty acid-binding protein 4 (FABP4, involved in fatty acid transport) and peroxisome proliferator-activated receptor alpha (PPARα, involved in lipid oxidation regulation) confirmed that rebamipide activates lipolysis and oxidation in dWAT [29].

### 2.3. Rebamipide Induces Lipolysis in Mature Adipocytes

**Figure 3:**
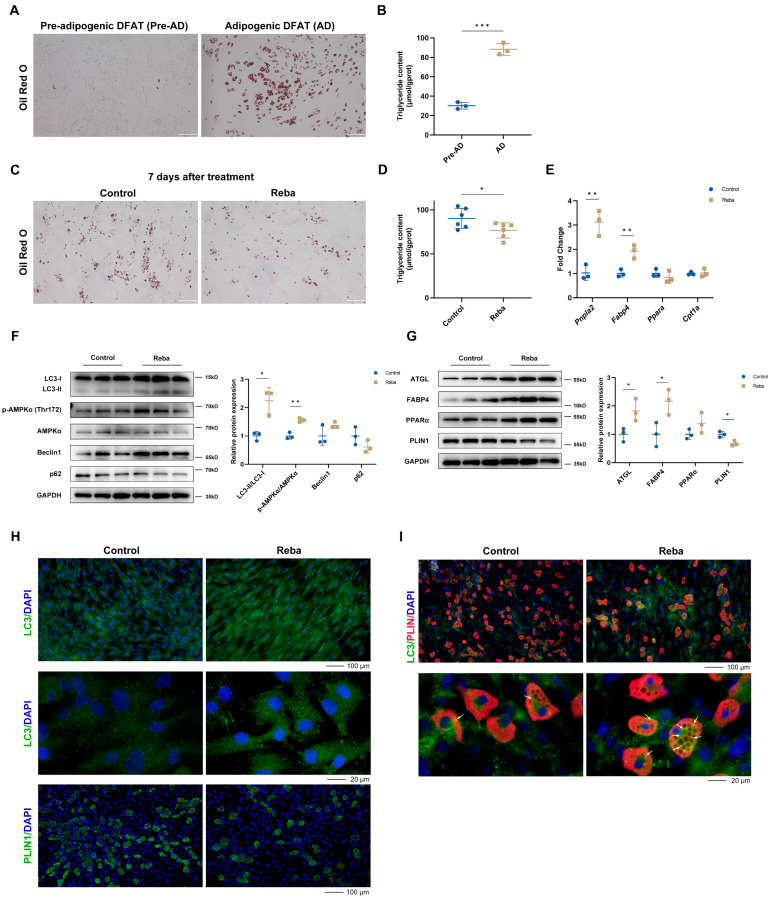
Rebamipide induces lipolysis in mature adipocytes. (**A**,**B**) Adipogenic induction of DFAT cells. Seven days after induction with differentiation medium, DFAT cells accumulated distinct lipid droplets, stained by Oil Red O (**A**), and quantified by triglyceride assay (**B**, n = 3 for each group). Scale bars, 200 μm. Data are presented as means ± SD, ***p < 0.001 compared with the pre-adipogenic DFAT cell group. (**C**,**D**) After adipogenic differentiation, DFAT cells were treated with DMSO (Control) or rebamipide (Reba) for 7 days prior to harvesting for molecular biology analysis. Oil Red O staining (**C**) and triglyceride assay (**D**) demonstrated that rebamipide induced lipolysis in mature adipocytes (n = 6 for each group). Scale bars, 200 μm. (**E**) Expression of lipolysis-related genes in differentiated DFAT cells, measured by qPCR (n = 3 for each group). (**F**,**G**) Western blot analysis of autophagy-related proteins (**F**) and lipolysis-related proteins (**G**) in differentiated DFAT cells (n = 3 for each group). (**H**) Immunofluorescence of LC3 and PLIN1 in differentiated DFAT cells. Nuclei were stained with DAPI (blue). Scale bar, 20 or 100 μm as indicated. (**I**) Double-labeled immunofluorescence of LC3 (green) and PLIN1 (red) in differentiated DFAT cells using the Tyramide Signal Amplification (TSA) kit. Nuclei were stained with DAPI (blue). Arrows indicate co-localized signals. Scale bar, 20 or 100 μm as indicated. Data are presented as means ± SD, * *p* < 0.05 and ** *p* < 0.01 compared with the control group.

To minimize interference from heterogeneous cell populations in skin tissue, we generated dedifferentiated fat (DFAT) cells via the ceiling culture method, yielding fibroblast-like multipotent adipocyte precursors [11] (Figure S1A). These DFAT cells expressed markers of adipocyte precursors (e.g., Thy1, Itgb1, Pdgfra) but low levels of genes involved in lipid metabolism (e.g., Fabp4, Adipoq, Pparg) [11] (Table S1, Figure S1B,C). After adipogenic induction, DFAT cells successfully differentiated into mature adipocytes, characterized by the accumulation of large, unilocular lipid droplets, elevated TG content, and upregulation of key lipid metabolism genes (Figure 3A,B, Table S1). This in vitro model enabled us to isolate the direct, cell-autonomous effects of rebamipide on adipocytes, separate from the complex paracrine signaling present in intact skin.

Treatment with rebamipide (20–200 μM) did not affect cell viability, indicating its favorable safety profile (Figure S1D). To determine the effects of rebamipide on adipocyte adipogenesis and lipolysis, DFAT cells were treated with 100 μM rebamipide for 7 days, either during or after adipogenic differentiation. Notably, although rebamipide did not affect adipogenesis (Figure S1E), it significantly reduced intracellular TG content in the differentiated DFAT cells (Figure 3C,D). Consistent with our in vivo findings, rebamipide activated autophagy through AMPK phosphorylation (Figure 3F) and upregulated the expression of lipolysis-related genes and proteins (Figure 3E,G). IF staining showed increased fluorescence intensity of LC3 puncta and reduced numbers of lipid droplets (Figure 3H). To further investigate the relationship between autophagy and lipolysis, differentiated DFAT cells were subjected to double-labeled immunofluorescence staining for LC3 and PLIN1. Tyramide signal amplification (TSA)-based detection demonstrated that rebamipide treatment significantly enhanced the co-localization of LC3 puncta with PLIN1 (Figure 3I), suggesting that rebamipide-induced lipolysis is coupled with enhanced autophagic activity in mature adipocytes.

### 2.4. Rebamipide-Induced Hair Regeneration Is Reversed by Lipid Metabolism Regulators

**Figure 4:**
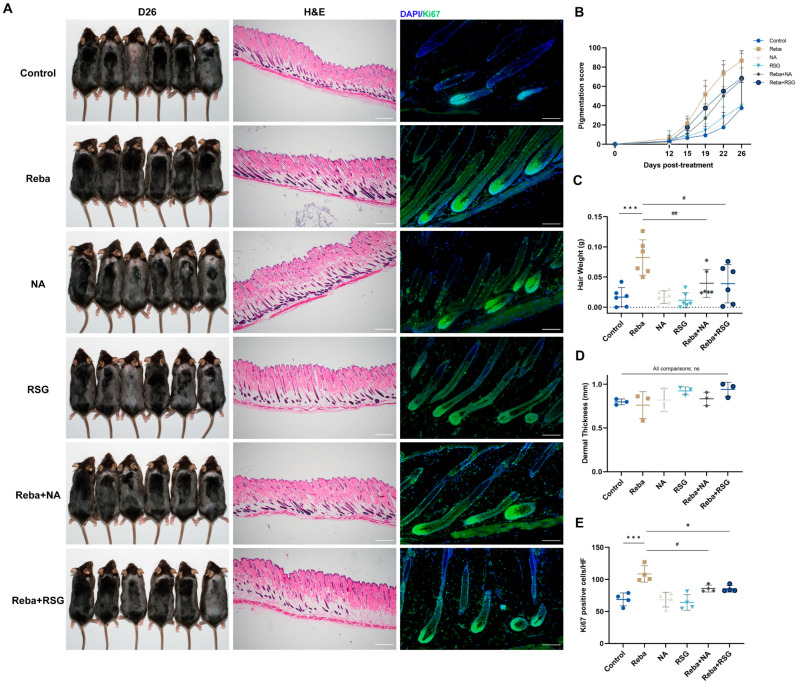
Rebamipide-induced hair regeneration is inhibited by niacin and rosiglitazone. (**A**) Seven-week-old male C57BL/6 mice were shaved and treated topically with vehicle, 3% rebamipide, 0.5% niacin (NA, lipolysis inhibitor), 0.05% rosiglitazone (RSG, PPARγ agonist), or combination therapies for 26 days (n = 6 for each group). Photographs were taken on day 26 post-treatment. H&E staining of skin sections revealed hair follicle morphology. Ki67 immunofluorescent staining showed that rebamipide-induced hair regeneration was inhibited by NA and RSG. Scale bars represent H&E staining, 500 μm; Ki67 staining, 100 μm. (**B**) Quantification of melanin pigmentation appearance (n = 6 for each group). (**C**) Quantification of regrown hair weight (n = 6 for each group). (**D**) Quantification of dermal thickness (n = 3 for each group). (**E**) Quantification of Ki67-positive cells per hair follicle (n = 4 for each group). Data are presented as means ± SD, ns, not significant, *** *p* < 0.001 compared with the control group. # *p* < 0.05 and ## *p* < 0.01 relative to the rebamipide-treated group.

To verify the hypothesis that rebamipide stimulates hair regeneration through the induction of dermal adipocyte lipolysis, we combined rebamipide with niacin (NA), a lipolysis inhibitor, or rosiglitazone (RSG), a PPARγ agonist that induces dWAT expansion [30]. Seven-week-old male C57BL/6 mice were shaved and divided into six treatment groups receiving daily vehicle control, 3% rebamipide, 0.5%NA (in drinking water), 0.05% RSG, rebamipide + NA, or rebamipide + RSG. Rebamipide alone significantly accelerated hair regeneration, as evidenced by increased pigmentation scores and the weight of regrown hair, while neither NA nor RSG alone had any significant effect. Notably, co-treatment with either NA or RSG partially reversed the effects of rebamipide (Figure 4A–C). Dorsal skin thickness did not differ significantly among the treatment groups (Figure 4D), likely due to rebamipide accelerating hair cycle progression into the telogen phase, resulting in dermal thinning. Quantification of Ki67+ cells in HF revealed that combined treatment significantly suppressed cell proliferation (Figure 4E), indicating that the effect of rebamipide on anagen induction is mediated by adipocyte lipolysis.

### 2.5. Rebamipide Induces Adipocyte Dedifferentiation and Activates HFSCs Through Growth Factor Secretion

**Figure 5:**
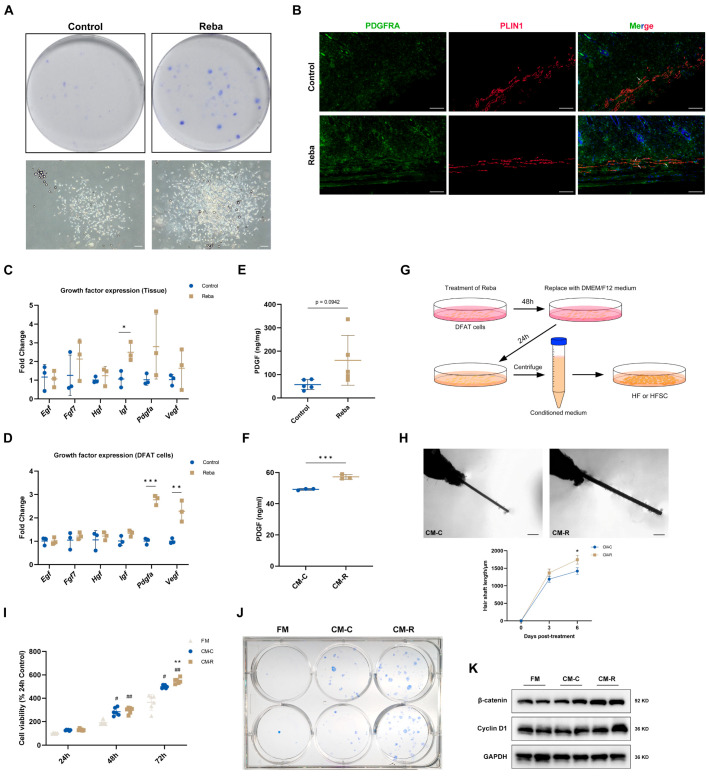
Rebamipide promotes adipocyte dedifferentiation and paracrine growth factor secretion to activate HFSCs. (**A**) Representative colony images of rebamipide-treated mature adipocytes during ceiling culture for 7 days. Scale bars, 500 μm. (**B**) Double-labeled immunofluorescence of skin sections for PDGFRA and PLIN1. Arrows indicate colocalized signals. Scale bars, 100 μm. (**C**,**D**) Differentiated DFAT cells were treated with rebamipide or DMSO for 48 h, followed by serum-free medium replacement and continued culture for 24 h. The culture supernatants were collected as conditioned medium (CM). Expression of growth factor-related genes in telogen skin (**C**) and differentiated DFAT cells (**D**) was analyzed by qPCR (n = 3 for each group). (**E**,**F**) Quantification of PDGF in skin extracts (n = 5 for each group) and CM (n = 3 for each group). (**G**) Schematic of CM preparation and functional assays. (**H**) Hair organ culture assays. Isolated mouse hair follicles were cultured with CM for 6 days (n = 16 for each group). Scale bars, 500 μm. (**I**) The viability of HFSCs treated with CM or fresh medium (FM) for 24, 48, and 72 h was assessed using the CCK-8 assay. Data are presented from 6 technical replicates. (**J**) Colony formation of HFSCs treated with CM or FM. Colonies were stained with Giemsa. (**K**) Western blot analysis of β-catenin and cyclin D1 in HFSCs treated with CM or FM for 7 days. Data are presented as means ± SD, * *p* < 0.05, ** *p* < 0.01, and *** *p* < 0.001 compared with the control group. # *p* < 0.05 and ## *p* < 0.01 relative to the fresh medium-treated group.

Previous studies have shown that lipolysis triggers the dedifferentiation of mature adipocytes into fibroblast-like cells [31,32,33]. Since rebamipide promoted lipolysis and reduced adipocyte size, we tested its potential for inducing adipocyte dedifferentiation. Mature adipocytes treated with 100 μM rebamipide during ceiling culture demonstrated a significant increase in colony formation after 10 days (Figure 5A), indicating that rebamipide enhanced the efficiency of adipocyte dedifferentiation. This finding was further validated by elevated expression of PDGFRA (adipocyte precursor marker) and increased colocalization of PDGFRA with PLIN1 (mature adipocyte marker) in the skin (Figure 5B).

Adipocyte progenitors secrete various growth factors (e.g., FGF, VEGF, IGF, PDGF) that promote hair growth [34]. To investigate whether rebamipide modulates the secretion of these growth factors, we analyzed the expression of growth factor-related genes in rebamipide-treated skin tissue and differentiated DFAT cells. Compared to the control group, rebamipide treatment significantly upregulated *Igf1*, *Vegfa* and *Pdgfa* (Figure 5C,D). Notably, *Pdgfa*, a known HF activator secreted by adipocyte precursors [6,8,9], showed the most pronounced changes. Elevated PDGF levels were confirmed by enzyme-linked immunosorbent assay (ELISA) in both treated skin and culture supernatants (Figure 5E,F).

To assess DFAT cell-mediated effects on HFs, conditioned medium (CM) from rebamipide-treated (CM-R) or DMSO-treated (CM-C) DFAT cells was applied to ex vivo HF culture (Figure 5G). HFs isolated from mouse whiskers were cultured with CM for 6 days in 48-well plates, and CM-R significantly improved HF viability and hair shaft elongation compared to CM-C (Figure 5H).

HFSCs in the bulge and hair germ become activated transiently at anagen onset to drive TAT [35]. To investigate the DFAT cell-mediated effects on HFSCs, rat vibrissae HFSCs were isolated and purified as previously described [36]. Briefly, bulges from vibrissae follicles were cultured on type IV collagen-coated dishes, allowing cells to migrate from the bulges and rapidly proliferate. HFSCs were then purified using the differential adherence method and characterized by their epithelial-like morphology (Figure S2A). qPCR analysis confirmed high expression of HFSC markers (*Itgb1*, *Itga6*, and *Krt15*) with minimal expression of the fibroblast marker Thy1 (Table S2). These findings were further validated at the protein level, with CD34 and integrin β1 expression confirmed by WB and IF analyses (Figures S1C and S2B).

Treatment with rebamipide (20–200 μM) did not affect cell viability in HFSCs, confirming its biological safety (Figure S2C). However, treatment with CM for 72 h significantly enhanced HFSC proliferation, with CM-R exhibiting stronger effects than CM-C (Figure 5I). Colony formation assays showed that CM-R increased the size and number of HFSC colonies, indicating that CM-R enhanced the stemness of HFSCs (Figure 5J). WB analysis revealed that CM-R significantly upregulated β-catenin and cyclin D1 expression in HFSCs (Figure 5K), suggesting that rebamipide-treated DFAT cells promote HFSC activation via paracrine factors.

### 2.6. Rebamipide Targets EP4 to Activate Autophagy and Lipolysis

Pharmacological studies have shown that prostaglandin E2 (PGE2), elevated in gastric tissue during rebamipide-driven gastric mucosal restitution [37], also promotes hair growth and prevents radiation-induced alopecia in mice [38,39]. However, our RNA-Seq and qPCR results showed that rebamipide did not alter PGE2 metabolic enzyme (Ptges and Hpgd) expression in skin (Figure S3A). PGE2 exerts its effects through four distinct E-type prostanoid (EP) receptors (EP1 to EP4), each with a unique tissue distribution pattern [40]. Notably, EP4, the predominant PGE2 receptor in the skin, was significantly more abundant in differentiated DFAT cells than in HFSCs (Figure S3B,C). Given that EP4 activation promotes both autophagosome formation [41] and lipolysis in WAT [42], we hypothesized that rebamipide directly binds to EP4 to promote hair regeneration.

**Figure 6:**
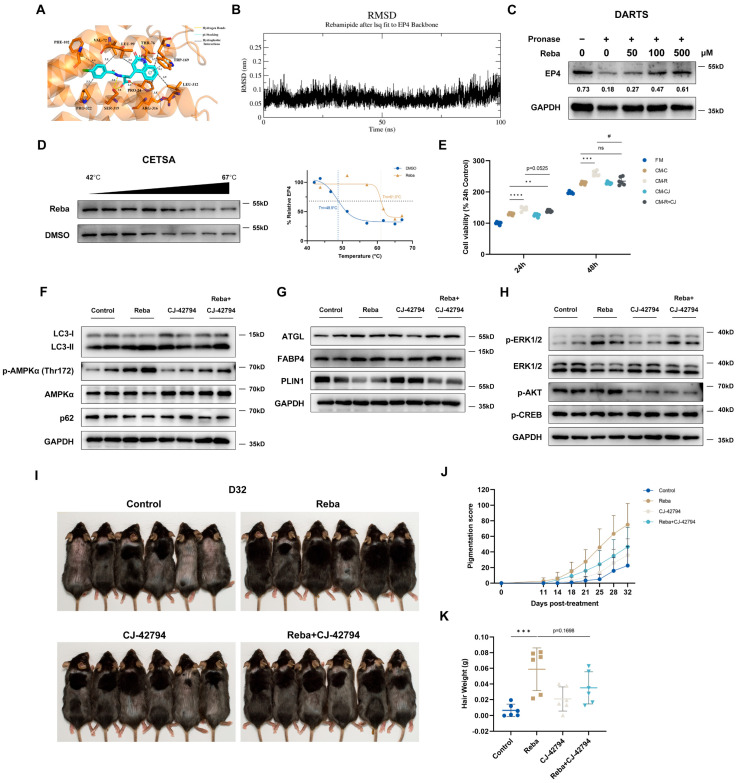
Rebamipide targets EP4 to activate autophagy and lipolysis. (**A**) Side view of rebamipide in the EP4 (PDB: 8GCP) ligand-binding site. Side chain interactions are shown in stick representation, highlighting the binding configuration. (**B**) The root mean squared deviation (RMSD) of rebamipide from 100 ns Molecular Dynamics (MD) simulations, calculated based on the initial complex state after 10 ns equilibration. The fluctuation of RMSD over time provides an indication of system stability. (**C**) Drug affinity-responsive target stability (DARTs) assays were performed by incubating DFAT cell lysates with rebamipide (0–500 μmol/L), followed by limited pronase digestion. Protein stability was then assessed by Western blot analysis of EP4 and loading controls. (**D**) Cellular thermal shift assay (CETSA) in intact DFAT cells was performed to evaluate the effect of rebamipide on EP4 thermal stability under temperatures ranging from 42 to 67 °C. (**E**) HFSCs were treated with FM or CM from DFAT cells pretreated with DMSO (CM-C), 100 μM rebamipide (CM-R), 100 nM CJ-42794 (CM-CJ), or their combination (CM-R+CJ). The viability of HFSCs was assessed after treatment with FM or CM for 24 and 48 h using the CCK-8 assay. Data are presented from 6 technical replicates. ns, not significant, ** *p* < 0.01, *** *p* < 0.001, and **** *p* < 0.0001 compared with the CM-C-treated group, # *p* < 0.05 relative to the CM-R-treated group. (**F**,**G**) Western blot analysis of autophagy-related proteins (**F**) and lipolysis-related proteins (**G**) in differentiated DFAT cells after 7 days of treatment with DMSO, rebamipide, CJ-42794, or their combination. (**H**) Western blot analysis of key phosphorylated proteins involved in EP4 downstream signaling in differentiated DFAT cells after 7 days of treatment. (**I**) Seven-week-old male C57BL/6 mice were shaved and treated with vehicle, 3% rebamipide, 0.05% CJ-42794, or their combination for 32 days (n = 6 for each group). Photographs were taken on day 32 post-treatment. (**J**) Quantification of melanin pigmentation appearance (n = 6 for each group). (**K**) Quantification of regrown hair weight (n = 6 for each group). Data are presented as means ± SD, *** *p* < 0.001 compared with the control group.

To confirm this hypothesis, molecular docking and binding energy calculations were performed using AutoDock Vina. The docking results revealed that rebamipide bound to EP4 (PDB: 8GCP) with a binding affinity of −9.53 kcal/mol. Additionally, rebamipide formed hydrogen bonds with residues Pro24 (3.6Å), Thr76 (3.0Å), Ser319 (2.8Å, 3.0Å) of EP4, a pi-stacking interaction with residue Trp169 (5.1Å), and hydrophobic interactions with 8 residues (Figure 6A). The interaction pattern with EP4 closely resembled that of endogenous PGE2 [43]. We then performed 100 ns molecular dynamics (MD) simulations of the rebamipide–EP4 complex. The root mean squared deviation (RMSD) of rebamipide, based on the initial complex state after 10 ns equilibration, fluctuated around 0.8Å from 0 to 100 ns, indicating system stability (Figure 6B). The simulations also revealed an average of 5 hydrogen bonds between rebamipide and EP4 (Figure S4), with an MM-PBSA binding energy of −60.21 kJ/mol (Table S3), further supporting the structural stability of the system.

The drug affinity-responsive target stability (DARTS) experiment, a widely used target validation technique, revealed that rebamipide dose-dependently increased EP4 content after protease treatment, indicating enhanced EP4 stability (Figure 6C). Similarly, the cellular thermal shift assay (CETSA) showed a significant melting temperature shift from 48.9 °C (DMSO) to 61.0 °C (rebamipide), further validating the rebamipide-mediated thermal stabilization of EP4 (Figure 6D).

To evaluate the role of EP4 activation in rebamipide-induced effects, we tested CJ-42794, a specific EP4 antagonist, on CM-mediated HFSC activation and autophagy/lipolysis in differentiated DFAT cells. CM from DFAT cells pre-treated with rebamipide and CJ-42794 significantly reduced HFSC proliferation compared to CM-R (Figure 6E). In addition, while CJ-42794 had minimal effects on autophagy and lipolysis, it partially reversed rebamipide-induced activation, especially the downregulation of p-AMPK, a key regulator of these processes [44] (Figure 6F,G). The EP4 receptor, a G protein-coupled receptor, stimulates adenylate cyclase to increase cyclic adenosine monophosphate (cAMP) levels, activating cAMP-responsive element-binding protein (CREB) via protein kinase A (PKA), and also triggers the PI3K/ERK and PI3K/Akt pathways [40,45]. WB analysis revealed that rebamipide did not affect p-CREB or p-Akt expression but significantly activated ERK1/2 phosphorylation, with this activation reversed by CJ-42794 (Figure 6H). Interestingly, EP4 coupled to the inhibitory G-protein (Gi) leads to the activation of PI3K/ERK signaling [40,45], consistent with our docking and MD simulations using the EP4-Gi complex conformation. These findings suggest that rebamipide promotes autophagy and lipolysis via EP4-mediated PI3K/ERK activation.

Finally, to evaluate the importance of EP4 activation in rebamipide-induced hair regeneration, we tested CJ-42794 in a murine model. As expected, CJ-42794 attenuated rebamipide-induced hair regeneration, as evidenced by reduced hair growth scores and regrown hair weight compared to rebamipide alone, suggesting that rebamipide promotes hair regrowth through an EP4-mediated pathway (Figure 6I–K).

## 3. Discussion

**Figure 7:**
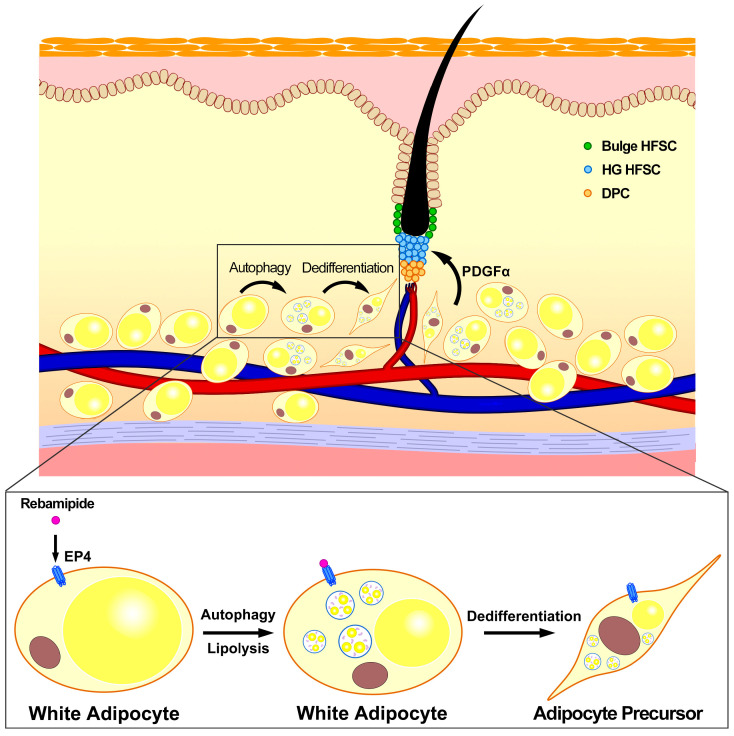
Schematic representation of the effects of rebamipide on dermal mature adipocytes. Rebamipide-induced autophagy and lipolysis in dermal mature adipocytes trigger their dedifferentiation into adipocyte precursors, which in turn promote the activation of HFSCs, potentially through increased PDGFα levels. HG: hair germ; DPC: dermal papilla cell.

Our study provides novel insights into the role of rebamipide in hair regeneration, highlighting its ability to activate the EP4 receptor-mediated signaling pathway in dermal adipocytes. We demonstrate that rebamipide induces autophagy and lipolysis in dermal white adipose tissue (dWAT), triggering adipocyte dedifferentiation and subsequently activating hair follicle stem cells (HFSCs), potentially through elevated PDGFα levels (Figure 7). Collectively, these findings not only position rebamipide as a unique therapeutic candidate that links adipocyte metabolic remodeling to HFSC activation but also identify EP4 as a novel therapeutic target for hair loss, offering a new direction for future research.

Although autophagy activation by small molecules stimulates hair regeneration, the spatial distribution of autophagic flux across skin remains poorly characterized due to its structural heterogeneity and complexity. By analyzing the distribution of LC3 in skin with IF, we found that autophagy is predominantly localized in dWAT, a highly plastic skin compartment. Interestingly, dermal adipocytes actively participate in diverse physiological processes, including hair cycling and wound healing [30]. Dermal adipocyte ablation accelerates HF reentry into anagen, while high-fat diet (HFD)-induced expansion delays this process. Additionally, recent evidence suggests that mature adipocytes retain the potential to dedifferentiate into adipocyte precursors during lactation [46], and lipolysis-mediated dedifferentiation into myofibroblasts is crucial for wound healing [31]. Based on these findings, our study demonstrates that rebamipide exerts three distinct biological effects: (1) inducing dermal adipocyte lipolysis, (2) promoting adipocyte dedifferentiation, and (3) subsequently activating HFSCs via elevated growth factor levels.

Our findings extend the understanding of dermal adipocyte biology in hair cycle regulation. Dermal adipocytes utilize autophagy machinery for lipolysis during the anagen–catagen transition [47]. This study reveals a new role for autophagy-mediated lipolysis in TAT, which aligns with previous studies showing that physiological stimuli like exercise, cold exposure, and fasting activate lipolysis [48] and also promote hair growth. Since cold exposure activates HFSCs via synapse-like contacts and norepinephrine [35], we propose that lipid metabolism mediates hair regrowth during cold exposure. Similarly, caloric restriction promotes hair growth through metabolic reprogramming in the dermis and reduces dWAT thickness [49]. Paradoxically, intermittent fasting triggers rapid lipolysis in the HFSC niche, releasing excessive free fatty acids (FFAs) that induce HFSC apoptosis [50]. Although intermittent fasting activates a comparable number of HFSCs, it simultaneously drives apoptosis through elevated intracellular reactive oxygen species (ROS) levels, which can be reversed by enhancing antioxidant capacity. These findings suggest that lipolysis requires the effective clearance of FFAs and ROS to maintain HFSC viability. Our study shows that rebamipide moderately induces lipolysis and enhances FFA oxidative phosphorylation. This effect preferentially promotes HFSC activation over apoptosis, demonstrating its superior ability to coordinate lipid mobilization and cellular redox homeostasis.

Originally developed for gastric ulcer therapy, rebamipide protects the mucosa via AMPK-mediated upregulation of gastric COX-2 and PGE2 [51] and alleviates gastric epithelial cell injury via ERK-dependent autophagy [19]. Here, we reveal a previously unrecognized role of rebamipide in promoting hair regeneration through PGE2-independent pathways in the skin. Silico simulations combined with target validation confirm the direct interaction of rebamipide with EP4. EP4 is linked to ERK-mediated autophagy in hepatic stellate cell activation via the PGE2/EP4 axis [52], and lipophagy in renal macrophages, which impedes the progression of chronic kidney disease [53]. Interestingly, EP4 regulates lipolysis through various mechanisms. Studies indicate that the PGE2-EP4 pathway facilitates lipolysis in WAT by enhancing *Pnpla2* transcription [42], and EP4 antagonists inhibit preadipocyte differentiation via the cAMP-PKA pathway [54]. However, EP4 knockout in mice leads to a reduction in body weight but disrupts lipid metabolism due to impaired TG clearance [55], highlighting its essential role in lipid homeostasis.

Beyond its mechanistic insights, our study highlights the considerable translational potential of rebamipide for treating hair loss disorders. A key advantage is its formulation for topical delivery, which we have demonstrated to be effective in promoting hair regrowth, with efficacy comparable to minoxidil, the current first-line topical therapy. Topical delivery offers several benefits, including localized treatment, reduced systemic side effects, and ease of application [56], making rebamipide a promising candidate for treating hair loss in a more targeted and safer manner. Importantly, rebamipide’s mechanism of action differs from minoxidil’s proposed vasodilatory effects or finasteride’s systemic hormonal modulation. This novel mechanism could offer significant benefit for patients who are unresponsive to existing therapies, with potential for combination strategies alongside other treatments. Moreover, rebamipide’s excellent safety profile [57,58], established through decades of clinical use for gastric ulcers and its subsequent repurposing for topical applications in ophthalmology and oral care, significantly de-risks its path to clinical evaluation for hair regeneration. This combination of novel targeting, topical efficacy, and a pre-established safety record makes rebamipide a uniquely promising and feasible candidate for rapid therapeutic development.

However, despite these promising findings, several limitations of our study should be acknowledged. First, the precise intracellular mechanisms downstream of EP4 that regulate adipose tissue metabolism need further investigation. Second, the temporal coordination between adipocyte lipolysis and HFSC activation requires more detailed analysis in vivo. Additionally, to validate EP4 as a therapeutic target, more comprehensive research is needed, including the use of novel EP4 agonists to confirm the observed phenotype. Furthermore, while our study clarifies the role of dWAT, it does not directly address the potential involvement of other follicular components, such as the dermal papilla. Future work should investigate their interaction with rebamipide to provide a more comprehensive understanding of the regenerative mechanism. Lastly, studies on human scalp tissue are essential to validate the translational relevance of the EP4–adipocyte axis, which would solidify EP4 as a therapeutic target and refine this novel strategy for treating hair loss.

## 4. Materials and Methods

### 4.1. Materials

Rebamipide (HY-B0360), niacin (HY-B0143), rosiglitazone (HY-17386), and CJ-42794 (HY-10797) were purchased from MCE (Monmouth Junction, NJ, USA); Collagen Type IV (C5533), collagenase type I (C0130), IBMX (I5879) were purchased from Sigma-Aldrich (St. Louis, MO, USA); culture media DMEM (KGL1206), DMEM/F12 (KGL1201) were obtained from KeyGEN BioTECH (Nanjing, China); fetal bovine serum (ST30-3302p) was from PAN Biotech (Aidenbach, Germany); EGF (C029) was from Novoprotein (Shanghai, China); hydrocortisone (40109ES08) and dexamethasone (40323ES25) were from YEASEN (Shanghai, China).

### 4.2. Animals

Wildtype male seven-week-old C57BL/6 mice were obtained from Ziyuan Experimental Animal Technology, and six-week-old Wistar rats were obtained from the College of Veterinary Medicine, Yangzhou University. Animals were housed in ventilated cages under 12 h light/dark cycles, with access to water and chow ad libitum. All experimental protocols were approved by the Animal Experimentation Ethics Committee of China Pharmaceutical University and adhered to the standards of the National Institutes of Health (NIH).

### 4.3. Topical Treatments

To systematically evaluate the hair regeneration potential and mechanism of rebamipide, seven-week-old male C57BL/6 mice in the telogen phase were dorsally shaved and randomized into treatment groups for separate experiments. Mice received daily topical treatments for 26–32 days as follows:

Experiment 1 (Dose–response): Vehicle (30% dipropylene glycol/70% water, homogenized for 1 min), 1% rebamipide, 3% rebamipide, or 2% minoxidil as a near-equimolar positive control.

Experiment 2 (Lipolysis Inhibition): Vehicle, 3% rebamipide, 0.05% rosiglitazone (a PPARγ agonist), 0.5% niacin (a lipolysis inhibitor) in drinking water, or their indicated combinations.

Experiment 3 (EP4 Antagonism): Vehicle, 3% rebamipide, 0.05% CJ-42794 (an EP4 antagonist), or their combination.

Hair growth was blindly scored based on pigmentation levels and hair shaft density [13]. At the endpoint, mice were anesthetized with 2.5% isoflurane for the collection of regrown hair and dorsal skin samples for subsequent analyses, including regrown hair weight measurement, dorsal skin histology, and molecular biology assays.

To investigate the underlying mechanisms, a separate cohort of mice was treated daily for 7 days with either vehicle or 3% rebamipide alone, following the same topical application protocol. Mice were sacrificed on day 7 for molecular biology analyses.

### 4.4. Histology and Immunofluorescent Staining

Mice were euthanized by cervical dislocation, and the shaved dorsal skin (4 × 2 cm^2^) was surgically excised along the hair growth axis. A central 2 × 1 cm^2^ full-thickness skin segment was fixed in 4% paraformaldehyde (PFA) at 4 °C for 24–48 h, while adjacent tissues were dissected into 20–40 mg fragments for molecular biology assays. The fixed samples were dehydrated through a graded ethanol series (70% to 100%), cleared in xylene, and embedded in paraffin using standard protocols. Serial sections (5 μm thickness) were cut perpendicularly to the skin surface to ensure consistent follicle orientation. Paraffin-embedded sections were dewaxed and hydrated. For H&E staining, sections were processed directly. For all immunofluorescence (IF) staining, antigen retrieval was performed by incubating the sections in Tris-EDTA buffer (pH 9.0) at 95 °C for 20 min in a water bath. Subsequently, sections were permeabilized, blocked, and incubated overnight at 4 °C with primary antibodies. The primary antibodies were used to detect specific targets as follows: anti-Ki67 (1:500; ab16667, Abcam, Cambridge, UK) to assess cell proliferation; anti-LC3 (1:200; 14600-1-AP, Proteintech, Wuhan, China) to monitor autophagy activation; anti-Perilipin 1 (1:500; 9349, CST, Danvers, MA, USA) to visualize lipid droplets and evaluate adipocyte morphology; anti-ATGL (1:200; 2439, CST) to confirm the induction of lipolysis; anti-Integrin β1 (1:200; SA40-08, Huabio, Hangzhou, China) and anti-CD34 (1:100; SI16-01, Huabio) as markers for HFSCs; and anti-PDGFRα (1:200; JF104-6, Huabio) to identify adipocyte precursors. Sections were then incubated with anti-rabbit Alexa Fluor 488 (1:500; ab150073, Abcam) secondary antibody for 1.5 h at room temperature. Nuclei were stained with DAPI to facilitate the localization of antigens within the tissue. After mounting, the slides were imaged with Olympus IX73 fluorescence microscopy, allowing for visualization of the fluorescence signals corresponding to the target proteins. For immunocytochemistry, cells were fixed in ice-cold 100% methanol and blocked for 30 min. Subsequent steps followed the protocol described above for tissue sections.

### 4.5. RNA-Seq

For RNA-seq analysis, an independent cohort of mice (n = 3 per group), separate from those used in other molecular biology assays, was treated topically with 3% rebamipide or vehicle for 7 days as outlined in Section 4.3. Total RNA was isolated from 200 mg of dorsal skin from three mice treated with rebamipide or vehicle using Trizol Reagent (Invitrogen, Carlsbad, CA, USA). RNA concentration, quality, and integrity were assessed using a NanoDrop spectrophotometer (Thermo, Waltham, MA, USA). Sequencing libraries were prepared by Shanghai Personal Biotechnology Co. Ltd. (http://www.personalbio.cn/ accessed on 15 October 2025) according to their standard protocols [59]. mRNA was purified from total RNA using poly-T oligo-attached magnetic beads and fragmented under elevated temperature in an Illumina proprietary buffer. First-strand cDNA was synthesized using random oligonucleotides and SuperScript II, followed by second-strand synthesis using DNA Polymerase I and RNase H. After blunt-ending and adenylation, Illumina adapters were ligated to the cDNA. Library fragments of 400–500 bp were selected using the AMPure XP system (Beckman Coulter, Beverly, CA, USA) and enriched by PCR. The final libraries were quantified and sequenced on the DNBSEQ-T7 platform.

For data analysis, the FPKM method was used to normalize gene expression levels. Differential expression analysis was performed using DESeq2 with thresholds set at |log2FoldChange| > 1 and a significant *p*-value < 0.05. Functional enrichment of the top 200 upregulated genes was conducted using the STRING database (https://string-db.org/ accessed on 15 October 2025) for KEGG and GO pathways. The Gene Set Enrichment Analysis (GSEA) (v4.1.0) tool was used for GSEA enrichment analysis of all genes.

### 4.6. RNA Isolation and Quantitative PCR

Total RNA was isolated using the RNA Isolation Kit (RC101, Vazyme, Nanjing, China). Reverse transcription (RT) was performed using oligo-dT primers (R223, Vazyme) with 1 μg of total RNA as template. qPCR analysis was performed using SYBR Green (Q312, Vazyme) on the Bio-Rad CFX96 System under the following conditions: 95 °C for 3 min, followed by 40 cycles of 95 °C for 10 s and 60 °C for 30 s. Gene expression levels were normalized to *Gapdh* and calculated using the 2^−ΔΔCt^ method. All primer sequences are summarized in Table S4.

### 4.7. Western Blot

Skin samples were lysed in cold RIPA buffer (P0013B, Beyotime, Shanghai, China) with protease (K1007, Apexbio, Houston, TX, USA) and phosphatase (K1015, Apexbio) inhibitors using a homogenizer (Jingxin, Shanghai, China). Lysates were incubated on ice for 30 min, then centrifuged at 12,000× *g* for 10 min. Supernatants were collected, and protein concentrations were measured using the BCA assay (E112, Vazyme). The lysates were boiled for 5 min in 4×Laemmli buffer (1610747, Bio-rad, Hercules, CA, USA), separated on 10% or 12% Tris-Glycine gels by SDS-PAGE, and transferred to PVDF membranes (1620177, Bio-rad) by wet transfer. Blots were probed with primary antibodies overnight at 4 °C: anti-β-Catenin (1:1000; 8480, CST), anti-Phospho-GSK3β (Ser9) (1:1000; 5558, CST), anti-GSK3β (1:5000; 12456, CST), anti-Cyclin D1 (1:5000; ET1601-31, Huabio), anti-LC3 (1:2000; 14600-1-AP, Proteintech), anti-p62 (1:5000; HA721171, Huabio), anti-Phospho-AMPKα (Thr172) (1:1000; 50081, CST), anti-AMPKα (1:1000; 5832, CST), anti-Beclin-1 (1:1000; 3495, CST), anti-CPT1A (1:1000; 12252, CST), anti-ATGL (1:1000; 2439, CST), anti-FABP4 (1:2000; 2120, CST), anti-PPARα (1:1000; ab126285, Abcam), anti-Integrin β1 (1:1000; SA40-08, Huabio), anti-CD34 (1:1000; SI16-01, Huabio), anti-PDGFRα (1:1000; JF104-6, Huabio), anti-EP4 (1:2000; 24895-1-AP, Proteintech) and anti-GAPDH (1:5000; 2118, CST), followed by incubation with anti-Rabbit IgG, HRP-linked antibody (7074, CST). The blots were then washed and scanned with the Bio-rad ChemiDoc Imaging System.

### 4.8. Double-Labeled Immunofluorescence

Paraffin-embedded skin tissue sections were first dewaxed and rehydrated to remove the paraffin and restore tissue hydration, which is essential for effective antibody binding. The sections were then incubated with Tris-EDTA buffer (pH 9.0) in a 95 °C water bath for 20 min to retrieve antigens. To reduce nonspecific binding, sections were blocked, then incubated overnight at 4 °C with Perilipin 1 antibody to target lipid droplets, followed by incubation with anti-rabbit HRP-conjugated secondary antibody for 50 min at room temperature. Signal amplification was performed using the Tyramide Signal Amplification (TSA) kit (G1259, Servicebio, Wuhan, China). Sections were incubated with the IF594-Tyramide dye for 10 min at room temperature. To thoroughly remove the antibodies from the first staining round, a second round of antigen retrieval and blocking was performed. Next, the sections were incubated sequentially with the LC3 antibody or PDGFRα antibody, followed by the secondary antibody and IF488-Tyramide, which provided a second fluorescent signal. Finally, nuclei were stained with DAPI to facilitate the localization of antigens within the tissue. After mounting, the slides were imaged with fluorescence microscopy, allowing for visualization of the fluorescence signals corresponding to the target proteins.

### 4.9. Primary Dedifferentiated Fat (DFAT) Cell Culture

Isolation of mature adipocytes and ceiling culture was performed under strict aseptic conditions as previously described [60], with successful dedifferentiation confirmed by the transition to a fibroblast-like morphology and the expression of progenitor markers. Briefly, inguinal fat samples from a Wistar rat were washed and minced, then digested in 0.1% collagenase type I at 37 °C for 1 h. Digestion releases individual mature adipocytes, allowing them to be separated from the stromal vascular fraction. The digested sample was filtered through a 250 µm filter and centrifuged at 150× *g* for 5 min. After two washes in complete medium (DMEM/F12 supplemented with 20% FBS), the floating adipocyte layer was transferred to culture dishes for ceiling culture. For dedifferentiation, dishes were inverted and maintained for 7 days. The dishes were then re-inverted, and the medium was changed to allow further growth of DFAT cells.

### 4.10. Primary Hair Follicle Stem Cell (HFSC) Culture

HFSCs isolation and purification were performed under strict aseptic conditions as previously described [36], with cell identity confirmed by the expression of stem cell markers. Briefly, vibrissa follicles were micro-dissected, and bulge segments were cultured in collagen IV-coated dishes with DMEM/F12 supplemented with 10% FBS, 20 ng/mL EGF, and 10 ng/mL hydrocortisone. On day 7, primary cells were digested with 0.125% trypsin for 7 min, with spindle cells discarded. Remaining cobblestone-like cells were further digested, centrifuged at 150× *g* for 5 min, and cultured in collagen IV-coated T25 flask for 20 min. The use of collagen IV leverages the rapid adhesion of stem cells to this matrix for enrichment. After discarding non-adherent cells, adherent cells were expanded, and the same purification procedure was repeated during the second passage.

### 4.11. Adipogenesis of DFAT Cells

Upon reaching confluence, cells were maintained in complete medium for 48 h to induce contact inhibition and allow the cells to exit the active cell cycle before differentiation induction. The differentiation medium (DMEM/F12 supplemented with 10% FBS, 10 μg/mL insulin, and 1 μM dexamethasone and 0.5 mM IBMX) was applied for 3 days, followed by maintenance medium (DMEM/F12 supplemented with 10% FBS and 10 μg/mL insulin), refreshed every 2 days. Lipid droplet accumulation became visible by light microscopy on day 7 and was confirmed by Oil Red O staining. For staining, cells were washed with PBS and fixed with 4% paraformaldehyde for 30 min at room temperature. After fixation, cells were stained with a freshly prepared and filtered Oil Red O working solution for 20 min, then sequentially washed with 60% isopropanol for 30 s and distilled water three times to remove non-specific staining.

### 4.12. Triglyceride and PDGF Assays

Triglyceride levels were measured using a Triglyceride Assay Kit (A110-1-1, Jiancheng, Nanjing, China) for tissue and cell culture samples as an indicator of cellular lipid content to assess lipid metabolism. Results were normalized to total protein content and expressed as μmol/g protein. Platelet-derived growth factor (PDGF), a key paracrine factor for HFSC activation, was measured using a PDGF ELISA Kit (ER1240, FineTest, Wuhan, China) for tissue and culture supernatant according to each manufacturer’s instructions. PDGF concentration was determined from a standard curve. For tissue samples, concentrations were normalized to total protein and are expressed as ng/mg protein. For culture supernatants, PDGF levels are expressed as ng/mL.

### 4.13. Conditioned Media Collection

Differentiated DFAT cells were treated with rebamipide (100 μM) or 0.1% DMSO for 48h. Cells were washed with PBS and incubated for 24 h with serum-free DMEM/F12 medium to avoid any interference from growth factors present in serum. Culture supernatants were then collected and filtered through 0.22 μm filters to eliminate any cellular debris. The samples were stored at −80 °C until further use.

### 4.14. HF Organ Culture

Hair follicle organ culture was performed under sterile conditions as previously described [61]. Briefly, vibrissa isolated from two C57BL/6 mice were washed with EBSS:PBS (1:1). Individual hair follicles were then carefully microdissected from the surrounding adipose and connective tissue under a stereomicroscope using fine forceps and micro-scissors, ensuring the integrity of the bulge and bulb regions. The isolated hair follicles were incubated in conditioned media supplemented with 10 μg/mL insulin at 37 °C, 5% CO_2_.

### 4.15. Cell Proliferation and Colony Formation Assays

DFAT cells and HFSCs were seeded in 96-well plates and treated with rebamipide or conditioned media. Cell proliferation rate was measured using the CCK-8 reagent (K1018, Apexbio) by absorbance at 450 nm after 2h incubation with a microplate reader (Tecan, Männedorf, Switzerland). For colony formation assays, HFSCs (800 cells/well) were cultured in 6-well plates with conditioned media supplemented with 5% FBS for 2 weeks to assess their long-term proliferation capacity and stem cell functionality. Cells were fixed in ice-cold methanol for 15 min, stained with Giemsa for 20 min and then washed with PBS to remove excess dye.

### 4.16. Molecular Docking

The atomic coordinates of EP4 were obtained from the Protein Data Bank (PDB ID: 8GCP), and the structure of rebamipide was obtained from PubChem. Both the protein and ligand were preprocessed. Molecular docking and binding affinity calculations were performed using the AutoDock Vina software (v1.2.5) [62]. The analysis of molecular interactions between rebamipide and EP4 was performed using PLIP [63].

### 4.17. Molecular Dynamics (MD) Simulation

The rebamipide–EP4 complex structure, docked by Vina, was embedded into a pre-equilibrated POPC membrane bilayer using input files generated from CHARMM-GUI. The system underwent energy minimization for 50,000 steps, followed by sequential NVT and NPT ensemble equilibrations in GROMACS 2020.6. A 10 ns equilibration phase preceded the final 100 ns MD simulation. All trajectory analyses were performed using GROMACS 2020.6. MM-PBSA binding free energy calculations were performed using the tool available at https://github.com/Jerkwin/gmxtools/blob/master/gmx_mmpbsa/gmx_mmpbsa.bsh (accessed on 15 October 2025).

### 4.18. Drug Affinity-Responsive Target Stability (DARTS) Experiment

DFAT cells were lysed in ice-cold RIPA buffer. The protein extracts were diluted in TNC buffer (50 mmol/L Tris-HCl, pH 8.0, 50 mmol/L NaCl, 10 mmol/L CaCl_2_) and treated with rebamipide (0–500 μM) for 1 h at room temperature to allow drug-target binding. Samples were then incubated with or without 0.3% pronase at room temperature for 15 min to subject unbound proteins to proteolysis. The reaction was stopped by the immediate addition of loading buffer and heat denaturation for EP4 level analysis by Western blot to detect ligand-induced protein stabilization.

### 4.19. Cellular Thermal Shift Assay (CETSA)

DFAT cells were pretreated with 100 μM rebamipide or 0.1% DMSO for 2 h before lysis in NP-40 buffer to allow drug-target engagement in a cellular context. Lysates were equally divided into 8 tubes and heated at gradually rising temperatures (from 42 to 67 °C; Bio-rad T-100) for 3 min to induce protein denaturation. After cooling, the samples were centrifuged at 12,000 rpm for 10 min at 4 °C, and the supernatants were collected for Western blot analysis to assess ligand-induced thermal stabilization of EP4.

### 4.20. Data and Statistical Analyses

Results are expressed as mean ± SD. Statistical analysis was performed using GraphPad Prism 9.5. The standard Student’s *t*-test or one-way ANOVA with Tukey’s post hoc test was performed for parametric data, while Welch’s correction was applied when variances were unequal. The Kruskal–Wallis test followed by Dunn’s post hoc was used for nonparametric data. Differences with *p* < 0.05 were considered statistically significant.

## 5. Conclusions

Our study identifies the gastric drug rebamipide as a promising agent for promoting hair regeneration through a novel mechanism. Rebamipide activates the EP4 receptor on dermal adipocytes, thereby triggering autophagy and enhancing lipolysis. This lipid metabolic remodeling drives adipocyte dedifferentiation and the subsequent secretion of PDGF-α, which activates dormant HFSCs to stimulate hair regrowth. Collectively, our findings not only position rebamipide as a potential repurposing candidate for treating hair loss disorders but also establish the EP4 receptor as a novel therapeutic target, highlighting the modulation of adipocyte metabolism as a viable strategy for treating hair loss.

## Data Availability

The data presented in this study are available on request from the corresponding author. The accession number for the raw and processed RNA-seq data reported in this manuscript is GEO: GSE296033.

## Supplementary Materials

The following supporting information can be downloaded at https://www.mdpi.com/article/10.3390/ijms262010132/s1.

## Abbreviations

The following abbreviations are used in this manuscript:

AC	Adenylate cyclase
AMPK	AMP-activated protein kinase
ATGL	Adipose triglyceride lipase
CETSA	Cellular thermal shift assay
CM	Conditioned medium
CPT1	Carnitine palmitoyltransferase 1
DARTS	Drug affinity-responsive target stability
DFAT cell	Dedifferentiated fat cell
dWAT	Dermal white adipose tissue
EP4	Prostaglandin E receptor 4
FABP4	Fatty acid-binding protein 4
HFSC	Hair follicle stem cell
PDGF	Platelet-derived growth factor
PGE2	Prostaglandin E2
PLIN1	Perilipin 1
TG	Triglyceride

## References

[1] Hamilton J.B. (1951). Patterned loss of hair in man; types and incidence. Ann. N. Y. Acad. Sci..

[2] Norwood O.T. (2001). Incidence of female androgenetic alopecia (female pattern alopecia). Dermatol. Surg. Off. Publ. Am. Soc. Dermatol. Surg..

[3] Wang T.L., Zhou C., Shen Y.W., Wang X.Y., Ding X.L., Tian S., Liu Y., Peng G.H., Xue S.Q., Zhou J.E. (2010). Prevalence of androgenetic alopecia in China: A community-based study in six cities. Br. J. Dermatol..

[4] Zhang C., Wang D., Wang J., Wang L., Qiu W., Kume T., Dowell R., Yi R. (2021). Escape of hair follicle stem cells causes stem cell exhaustion during aging. Nat. Aging.

[5] Price V.H. (1999). Treatment of hair loss. N. Engl. J. Med..

[6] Guerrero-Juarez C.F., Plikus M.V. (2018). Emerging nonmetabolic functions of skin fat. Nat. Rev. Endocrinol..

[7] Kruglikov I.L., Zhang Z., Scherer P.E. (2019). The Role of Immature and Mature Adipocytes in Hair Cycling. Trends Endocrinol. Metab..

[8] Festa E., Fretz J., Berry R., Schmidt B., Rodeheffer M., Horowitz M., Horsley V. (2011). Adipocyte lineage cells contribute to the skin stem cell niche to drive hair cycling. Cell.

[9] Zwick R.K., Guerrero-Juarez C.F., Horsley V., Plikus M.V. (2018). Anatomical, Physiological, and Functional Diversity of Adipose Tissue. Cell Metab..

[10] Song T., Kuang S. (2019). Adipocyte dedifferentiation in health and diseases. Clin. Sci..

[11] Shen J.F., Sugawara A., Yamashita J., Ogura H., Sato S. (2011). Dedifferentiated fat cells: An alternative source of adult multipotent cells from the adipose tissues. Int. J. Oral Sci..

[12] Dermitzakis I., Kampitsi D.D., Manthou M.E., Evangelidis P., Vakirlis E., Meditskou S., Theotokis P. (2024). Ontogeny of Skin Stem Cells and Molecular Underpinnings. Curr. Issues Mol. Biol..

[13] Chai M., Jiang M., Vergnes L., Fu X., de Barros S.C., Doan N.B., Huang W., Chu J., Jiao J., Herschman H. (2019). Stimulation of Hair Growth by Small Molecules that Activate Autophagy. Cell Rep..

[14] Manzoor M., Chen D., Lin J., Wang Y., Xiang L., Qi J. (2025). Isoquercitrin promotes hair growth through induction of autophagy and angiogenesis by targeting AMPK and IGF-1R. Phytomedicine.

[15] Kang J.I., Choi Y.K., Han S.C., Kim H.G., Hong S.W., Kim J., Kim J.H., Hyun J.W., Yoo E.S., Kang H.K. (2022). Limonin, a Component of Immature Citrus Fruits, Activates Anagen Signaling in Dermal Papilla Cells. Nutrients.

[16] Singh R., Kaushik S., Wang Y., Xiang Y., Novak I., Komatsu M., Tanaka K., Cuervo A.M., Czaja M.J. (2009). Autophagy regulates lipid metabolism. Nature.

[17] Pan J., Kothan S., Liu L., Moe A.T.M., Dong L., Sun Y., Yang Y. (2021). Autophagy participants in the dedifferentiation of mouse 3T3-L1 adipocytes triggered by hypofunction of insulin signaling. Cell Signal..

[18] Genta R.M. (2003). Review article: The role of rebamipide in the management of inflammatory disease of the gastrointestinal tract. Aliment. Pharmacol. Ther..

[19] He Q., Liu M., Rong Z., Liang H., Xu X., Sun S., Lei Y., Li P., Meng H., Zheng R. (2022). Rebamipide attenuates alcohol-induced gastric epithelial cell injury by inhibiting endoplasmic reticulum stress and activating autophagy-related proteins. Eur. J. Pharmacol..

[20] Hasegawa S., Sekino H., Matsuoka O., Saito K., Sekino H., Morikawa A., Uchida K., Koike M., Azuma J. (2003). Bioequivalence of rebamipide granules and tablets in healthy adult male volunteers. Clin. Drug Investig..

[21] Kinoshita S., Awamura S., Oshiden K., Nakamichi N., Suzuki H., Yokoi N., Rebamipide Ophthalmic Suspension Phase II Study Group (2012). Rebamipide (OPC-12759) in the treatment of dry eye: A randomized, double-masked, multicenter, placebo-controlled phase II study. Ophthalmology.

[22] Elsaadany B., Anayb S.M., Mashhour K., Yossif M., Zahran F. (2024). Rebamipide gargle and benzydamine gargle in prevention and management of chemo-radiotherapy and radiotherapy-induced oral mucositis in head and neck cancer patients (randomized clinical trial). BMC Oral Health.

[23] Jhun J., Kwon J.E., Kim S.Y., Jeong J.H., Na H.S., Kim E.K., Lee S.H., Jung K., Min J.K., Cho M.L. (2017). Rebamipide ameliorates atherosclerosis by controlling lipid metabolism and inflammation. PLoS ONE.

[24] Jhun J., Moon J., Kim S.Y., Cho K.H., Na H.S., Choi J., Jung Y.J., Song K.Y., Min J.K., Cho M.L. (2022). Rebamipide treatment ameliorates obesity phenotype by regulation of immune cells and adipocytes. PLoS ONE.

[25] Stenn K.S., Paus R. (2001). Controls of hair follicle cycling. Physiol. Rev..

[26] Deschene E.R., Myung P., Rompolas P., Zito G., Sun T.Y., Taketo M.M., Saotome I., Greco V. (2014). beta-Catenin activation regulates tissue growth non-cell autonomously in the hair stem cell niche. Science.

[27] Shaid S., Brandts C.H., Serve H., Dikic I. (2013). Ubiquitination and selective autophagy. Cell Death Differ..

[28] Zimmermann R., Strauss J.G., Haemmerle G., Schoiswohl G., Birner-Gruenberger R., Riederer M., Lass A., Neuberger G., Eisenhaber F., Hermetter A. (2004). Fat mobilization in adipose tissue is promoted by adipose triglyceride lipase. Science.

[29] Nakamura M.T., Yudell B.E., Loor J.J. (2014). Regulation of energy metabolism by long-chain fatty acids. Prog. Lipid Res..

[30] Zhang Z., Shao M., Hepler C., Zi Z., Zhao S., An Y.A., Zhu Y., Ghaben A.L., Wang M.Y., Li N. (2019). Dermal adipose tissue has high plasticity and undergoes reversible dedifferentiation in mice. J. Clin. Investig..

[31] Shook B.A., Wasko R.R., Mano O., Rutenberg-Schoenberg M., Rudolph M.C., Zirak B., Rivera-Gonzalez G.C., Lopez-Giraldez F., Zarini S., Rezza A. (2020). Dermal Adipocyte Lipolysis and Myofibroblast Conversion Are Required for Efficient Skin Repair. Cell Stem Cell.

[32] Simiczyjew A., Wadzynska J., Pietraszek-Gremplewicz K., Kot M., Zietek M., Matkowski R., Nowak D. (2023). Melanoma cells induce dedifferentiation and metabolic changes in adipocytes present in the tumor niche. Cell. Mol. Biol. Lett..

[33] Sun L., Zhang X., Wu S., Liu Y., Guerrero-Juarez C.F., Liu W., Huang J., Yao Q., Yin M., Li J. (2023). Dynamic interplay between IL-1 and WNT pathways in regulating dermal adipocyte lineage cells during skin development and wound regeneration. Cell Rep..

[34] Choi N., Shin S., Song S.U., Sung J.H. (2018). Minoxidil Promotes Hair Growth through Stimulation of Growth Factor Release from Adipose-Derived Stem Cells. Int. J. Mol. Sci..

[35] Shwartz Y., Gonzalez-Celeiro M., Chen C.L., Pasolli H.A., Sheu S.H., Fan S.M., Shamsi F., Assaad S., Lin E.T., Zhang B. (2020). Cell Types Promoting Goosebumps Form a Niche to Regulate Hair Follicle Stem Cells. Cell.

[36] Quan R., Zheng X., Ni Y., Xie S., Li C. (2016). Culture and characterization of rat hair follicle stem cells. Cytotechnology.

[37] Takahashi M., Takada H., Takagi K., Kataoka S., Soma R., Kuwayama H. (2003). Gastric restitution is inhibited by dexamethasone, which is reversed by hepatocyte growth factor and rebamipide. Aliment. Pharmacol. Ther..

[38] Cheng H., Liu F., Zhou M., Chen S., Huang H., Liu Y., Zhao X., Zhang Q., Zhou X., Li Z. (2022). Enhancement of hair growth through stimulation of hair follicle stem cells by prostaglandin E2 collagen matrix. Exp. Cell Res..

[39] Hanson W.R., Pelka A.E., Nelson A.K., Malkinson F.D. (1992). Subcutaneous or topical administration of 16,16 dimethyl prostaglandin E2 protects from radiation-induced alopecia in mice. Int. J. Radiat. Oncol. Biol. Phys..

[40] Konya V., Marsche G., Schuligoi R., Heinemann A. (2013). E-type prostanoid receptor 4 (EP4) in disease and therapy. Pharmacol. Ther..

[41] Nakamura K., Kageyama S., Ito T., Hirao H., Kadono K., Aziz A., Dery K.J., Everly M.J., Taura K., Uemoto S. (2019). Antibiotic pretreatment alleviates liver transplant damage in mice and humans. J. Clin. Investig..

[42] Inazumi T., Yamada K., Shirata N., Sato H., Taketomi Y., Morita K., Hohjoh H., Tsuchiya S., Oniki K., Watanabe T. (2020). Prostaglandin E(2)-EP4 Axis Promotes Lipolysis and Fibrosis in Adipose Tissue Leading to Ectopic Fat Deposition and Insulin Resistance. Cell Rep..

[43] Huang S.M., Xiong M.Y., Liu L., Mu J., Wang M.W., Jia Y.L., Cai K., Tie L., Zhang C., Cao S. (2023). Single hormone or synthetic agonist induces G(s)/G(i) coupling selectivity of EP receptors via distinct binding modes and propagating paths. Proc. Natl. Acad. Sci. USA.

[44] Trefts E., Shaw R.J. (2021). AMPK: Restoring metabolic homeostasis over space and time. Mol. Cell.

[45] Yokoyama U., Iwatsubo K., Umemura M., Fujita T., Ishikawa Y. (2013). The prostanoid EP4 receptor and its signaling pathway. Pharmacol. Rev..

[46] Wang Q.A., Song A., Chen W., Schwalie P.C., Zhang F., Vishvanath L., Jiang L., Ye R., Shao M., Tao C. (2018). Reversible De-differentiation of Mature White Adipocytes into Preadipocyte-like Precursors during Lactation. Cell Metab..

[47] Nicu C., Hardman J.A., Pople J., Paus R. (2019). Do human dermal adipocytes switch from lipogenesis in anagen to lipophagy and lipolysis during catagen in the human hair cycle?. Exp. Dermatol..

[48] Grabner G.F., Xie H., Schweiger M., Zechner R. (2021). Lipolysis: Cellular mechanisms for lipid mobilization from fat stores. Nat. Metab..

[49] Forni M.F., Peloggia J., Braga T.T., Chinchilla J.E.O., Shinohara J., Navas C.A., Camara N.O.S., Kowaltowski A.J. (2017). Caloric Restriction Promotes Structural and Metabolic Changes in the Skin. Cell Rep..

[50] Chen H., Liu C., Cui S., Xia Y., Zhang K., Cheng H., Peng J., Yu X., Li L., Yu H. (2025). Intermittent fasting triggers interorgan communication to suppress hair follicle regeneration. Cell.

[51] Lee S., Jeong S., Kim W., Kim D., Yang Y., Yoon J.H., Kim B.J., Min D.S., Jung Y. (2017). Rebamipide induces the gastric mucosal protective factor, cyclooxygenase-2, via activation of 5’-AMP-activated protein kinase. Biochem. Biophys. Res. Commun..

[52] Cao Y., Mai W., Li R., Deng S., Li L., Zhou Y., Qin Q., Zhang Y., Zhou X., Han M. (2022). Macrophages evoke autophagy of hepatic stellate cells to promote liver fibrosis in NAFLD mice via the PGE2/EP4 pathway. Cell. Mol. Life Sci..

[53] Guan X., Liu Y., Xin W., Qin S., Gong S., Xiao T., Zhang D., Li Y., Xiong J., Yang K. (2022). Activation of EP4 alleviates AKI-to-CKD transition through inducing CPT2-mediated lipophagy in renal macrophages. Front. Pharmacol..

[54] Sugimoto Y., Tsuboi H., Okuno Y., Tamba S., Tsuchiya S., Tsujimoto G., Ichikawa A. (2004). Microarray evaluation of EP4 receptor-mediated prostaglandin E2 suppression of 3T3-L1 adipocyte differentiation. Biochem. Biophys. Res. Commun..

[55] Cai Y., Ying F., Song E., Wang Y., Xu A., Vanhoutte P.M., Tang E.H. (2015). Mice lacking prostaglandin E receptor subtype 4 manifest disrupted lipid metabolism attributable to impaired triglyceride clearance. FASEB J..

[56] Zhao L., Chen J., Bai B., Song G., Zhang J., Yu H., Huang S., Wang Z., Lu G. (2023). Topical drug delivery strategies for enhancing drug effectiveness by skin barriers, drug delivery systems and individualized dosing. Front. Pharmacol..

[57] Kim J.H., Park S.H., Cho C.S., Lee S.T., Yoo W.H., Kim S.K., Kang Y.M., Rew J.S., Park Y.W., Lee S.K. (2014). Preventive efficacy and safety of rebamipide in nonsteroidal anti-inflammatory drug-induced mucosal toxicity. Gut Liver.

[58] Jang E., Park M., Jeong J.E., Lee J.Y., Kim M.G. (2022). Frequently reported adverse events of rebamipide compared to other drugs for peptic ulcer and gastroesophageal reflux disease. Sci. Rep..

[59] Zhou H., He J., Liu R., Cheng J., Yuan Y., Mao W., Zhou J., He H., Liu Q., Tan W. (2024). Microenvironment-responsive metal-phenolic network release platform with ROS scavenging, anti-pyroptosis, and ECM regeneration for intervertebral disc degeneration. Bioact. Mater..

[60] Wei S., Du M., Jiang Z., Duarte M.S., Fernyhough-Culver M., Albrecht E., Will K., Zan L., Hausman G.J., Elabd E.M. (2013). Bovine dedifferentiated adipose tissue (DFAT) cells: DFAT cell isolation. Adipocyte.

[61] Philpott M.P., Szallasi A., Bíró T. (2012). Hair Follicle Culture. TRP Channels in Drug Discovery: Volume II.

[62] Forli S., Huey R., Pique M.E., Sanner M.F., Goodsell D.S., Olson A.J. (2016). Computational protein-ligand docking and virtual drug screening with the AutoDock suite. Nat. Protoc..

[63] Adasme M.F., Linnemann K.L., Bolz S.N., Kaiser F., Salentin S., Haupt V.J., Schroeder M. (2021). PLIP 2021: Expanding the scope of the protein-ligand interaction profiler to DNA and RNA. Nucleic Acids Res.

