# Supplementary material for: Rebamipide Induces Hair Regeneration Through EP4-Driven Lipid Metabolism Remodeling
Source: Int J Mol Sci. 2025 Oct 18;26(20):10132. doi: 10.3390/ijms262010132 (PMC12562944; doi:10.3390/ijms262010132)
Supplement: Supplementary file 1 [file ijms-26-10132-s001.zip › ijms-3897468-supplementary.pdf]

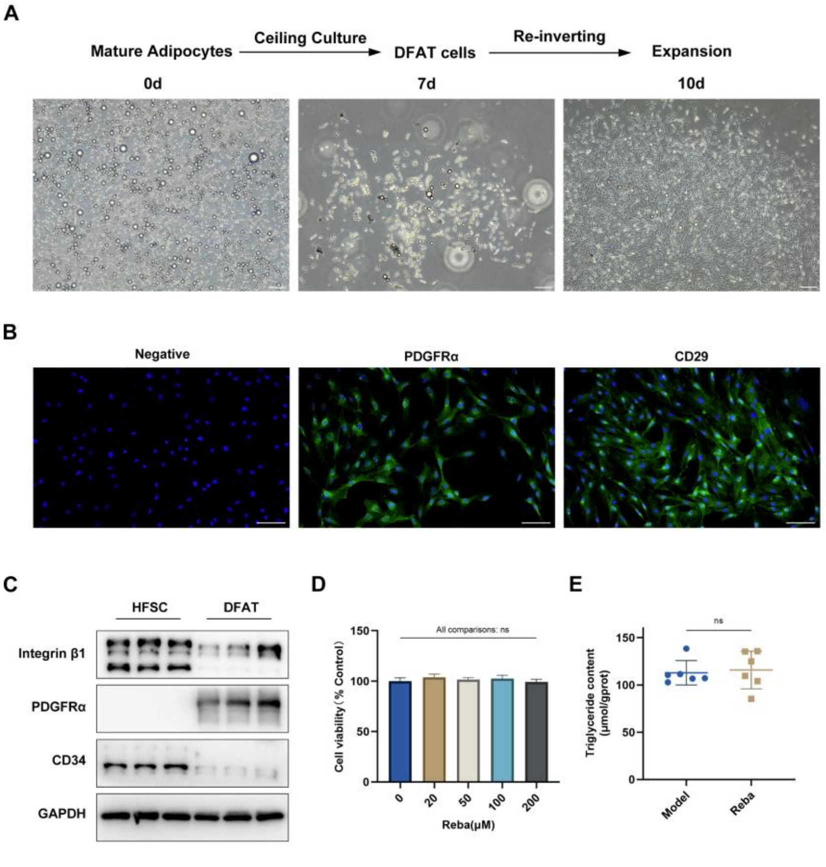

Figure S1. Establishment and identification of DFAT cells. (**A**) Mature adipocytes were isolated from inguinal white adipose tissue (iWAT) of rats by enzymatic digestion. Using ceiling culture for 7 days, the mature adipocytes dedifferentiated and attached to the ceiling of the plate. After re-inverting the plate, DFAT cells were further cultured for expansion. Scale bars, 500 μm. (**B**) Representative immunofluorescence images showing CD29 and PDGFRα expression in DFAT cells. Scale bars, 100 μm. (**C**) Western blot analysis of CD29, PDGFRα, and CD34 expression in DFAT cells and HFSCs. (**D**) Viability of DFAT cells assessed by CCK-8 assay after rebamipide treatment for 24 hours. Data are presented from 6 technical replicates. (**E**) During adipogenic differentiation, DFAT cells were treated with 0.1% DMSO or 100 μM rebamipide. The inhibitory effect of rebamipide on adipogenesis was assessed by intracellular triglyceride measurements (n = 6 for each group). Data are presented as means ± SD. ns, not significant.

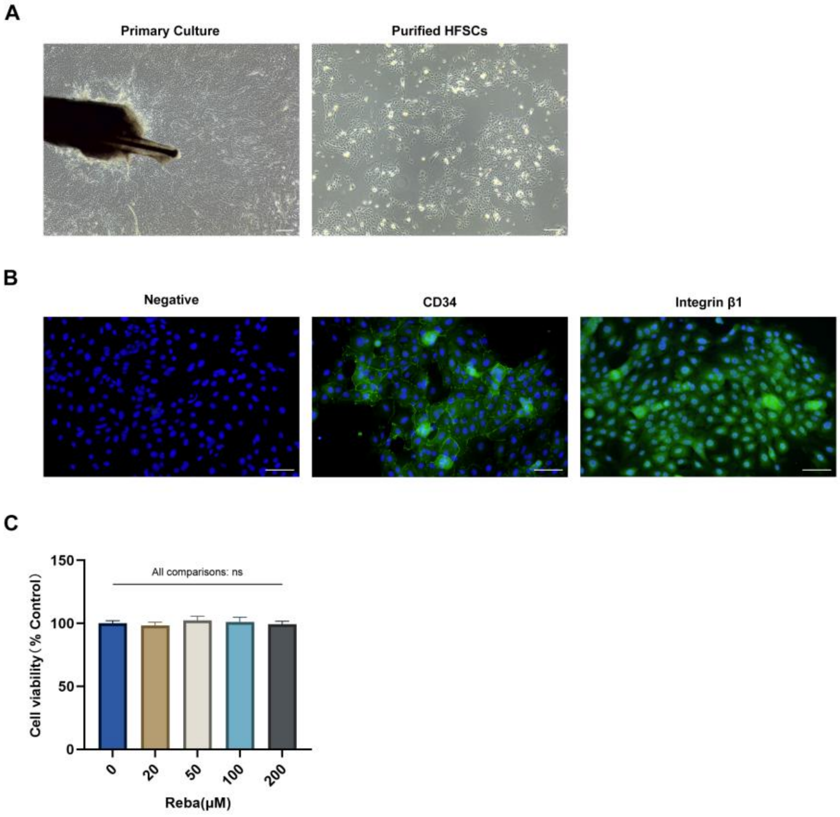

Figure S2. Establishment and identification of HFSCs. (**A**) Rat vibrissa follicles were isolated and cultured for 7days. HFSCs were purified using a type-IV collagen differential adherence method. Scale bars, 500 μm. (**B**) Immunofluorescence images showing CD29 and CD34 expression in HFSCs. Scale bars, 100 μm. (**C**) Viability of HFSCs assessed by CCK-8 assay after rebamipide treatment for 24 hours. Data are presented from 6 technical replicates. Data are presented as means ± SD. ns, not significant.

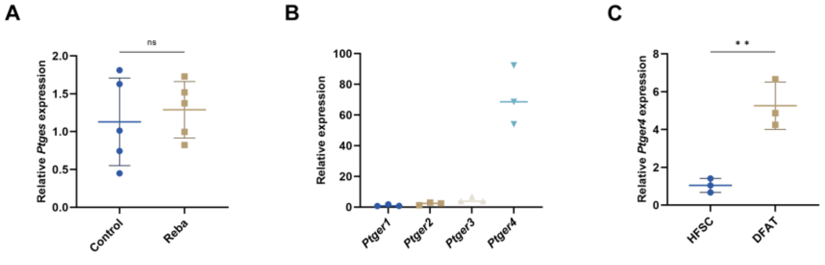

Figure S3. Expression analysis of Ptges and EP subtypes. (A) qPCR analysis of *Ptges* expression in the skin of mice treated with rebamipide or vehicle (n = 5 for each group). (B) qPCR analysis of the expression levels of four EP subtypes in mouse skin (n = 3 for each group). (C) qPCR analysis of *Ptger4* expression in HFSCs versus differentiated DFAT cells (n = 3 for each group). Data are presented as means ± SD. ns, not significant; **p<0.01 compared with HFSC.

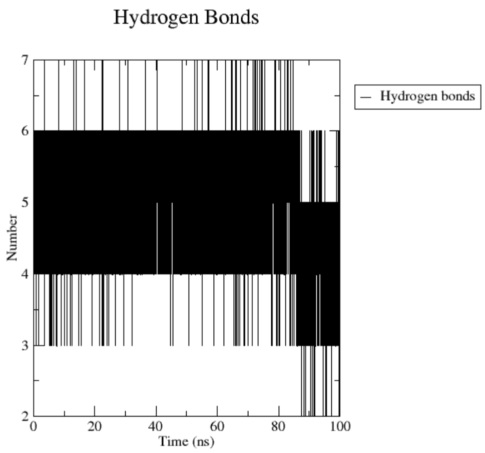

Figure S4. Stability of the rebamipide-EP4 complex during MD simulations. Dynamic changes in the number of hydrogen bonds formed between rebamipide and the EP4 receptor over the 100 ns simulation trajectory.

Table S1. Gene expression profile of DFAT cells before and after adipogenic differentiation. qPCR analysis of adipocyte precursor markers and mature adipocyte markers in pre-adipogenic and differentiated DFAT cells (n=3 for each group).

Gene	Pre-adipogenic DFAT cells		Differentiated DFAT cells	
	Average	△Ct	Average	△Ct
Thy1	25.18 ± 0.17	3.42 ± 0.16	25.12 ± 0.67	3.45 ± 0.52
Itgb1	24.04 ± 0.11	2.27 ± 0.08	24.39 ± 0.13	2.72 ± 0.02
Pdgfra	25.28 ± 0.31	3.51 ± 0.29	25.28 ± 0.11	3.61 ± 0.25
Fabp4	29.94 ± 0.28	8.18 ± 0.24	24.38 ± 0.86	2.71 ± 1.00***
Adipoq	30.04 ± 0.65	8.27 ± 0.63	24.95 ± 0.41	3.28 ± 0.54***
Pparg	31.09 ± 0.29	9.32 ± 0.29	28.22 ± 0.64	6.55 ± 0.79**
Gapdh	21.77 ± 0.03	-	21.67 ± 0.15	-

Data are presented as means ± SD, *p<0.05 and **p<0.01 compared with pre-adipogenic DFAT cell group.

Table S2. Gene expression profile of purified HFSCs. qPCR analysis of established HFSC markers in the isolated cell population (n=3 for each group).

Gene	Average	△Ct
Itgb1	24.00 ± 0.72	4.08 ± 0.26
Itga6	25.87 ± 0.71	5.96 ± 0.13
Krt15	28.98 ± 0.99	9.06 ± 0.80
Thy1	34.48 ± 1.66	14.56 ± 2.26
Gapdh	19.92 ± 0.63	-

Data are presented as means ± SD.

Table S3. MM-PBSA binding free energy analysis (kJ/mol).

Group	VDW	COU (with DH)	PB	SA	ΔH (with DH)	Entropy (-TΔS, with DH)	Binding Energy
Rebamipide -EP4	-152.065	-227.794	325.462	-26.578	-80.975	20.766	-60.209

Table S4. Sequence of primers used for qPCR.

Name	5′ primer	3′ primer
Pnpla2	GGACCTGATGACCACCCTTTC	AGCCACTCCAACAAGCGGA
Cpt1a	AAGAACATCGTGAGTGGCGT	ACCTTGACCATAGCCATCCAG
Fabp4	ATGAAAGAAGTGGGAGTTGGC	TTCCACGCCCAGTTTGAAGG
Ppara	ACCACTACGGAGTTCACGCATG	GAATCTTGCAGCTCCGATCACAC
Thy1	AAGAGGAGGCTGCAAGCTAGG	CTCGGGACATCTGCAAGACTGAA
Itgb1	CAAGTGGGACACGGGTGAAA	AGAGCCCCAAAGCTACCCTA
Pdgfra	GCACCAAGTCAGGTCCCATT	TGTCCAGGTCTTTCTTCGGC
Adipoq	CGCAGGTGTTCTTGGTCCTAAGG	GCCCTACGCTGAATGCTGAGTG
Pparg	GCTCCAAGAATACCAAAGTGCG	ACAGACTCGGCACTCAATGG
Itga6	ACAACGCCTTTCTTCGGCTA	CACTGGCCGGGATCTGAAAA
Krt15	GCCAGGATGCTAAGATGGCT	GCATGGACTGGAGTCTGCTT
Egf	ACGAACTTGTTTCCTGCCCA	TCAGGCGATGAACAACCAGT
Hgf	ACCTACAGGAAAACTACTGTCG	TGCATTCAACTTCTGAACACTG
Igf	CCTCTTCTACCTGGCGCTCT	ATAGCCTGTGGGCTTGTTGAAG
Vegfa	CCAAAGCCAGCACATAGGAGA	GTTCTGTCTTTCTTTGGTCTGC
Fgf7	GAGAGGCTCAAGTTGCACGA	CGGTTGCTCCTTGACTTTTGT
Pdgfa	CTGGCTCGAAGTCAGATCCACA	GACTTGTCTCCAAGGCATCCTC
Ptges	TGTACGGGTAGACCTCTATGAA	CTTTGCAGCTAGGGTTGACAT
Ptger1	TCATGGTGGTGTCGTGCATCTG	GTCCAGGATCTGGTTCCACGAT
Ptger2	GAGAGAGGACTTCGATGGCAGA	GGAAGAGGTTTCATCCATGTAGG
Ptger3	GCTTCGCTGAACCAGATCTTGG	CAGGTACTGCAATGAAAGTCCAC
Ptger4	GTCATCTTACTCATCGCCACCT	TAATGAACACTCGCACCACG
Gapdh	CATCACTGCCACCCAGAAGACTG	ATGCCAGTGAGCTTCCCGTTCAG
